# Parking space detection using computer vision: a systematic review of the literature

**DOI:** 10.3389/frai.2026.1854166

**Published:** 2026-08-24

**Authors:** Gary Fernando Yunganina Mamani, Guver Leon Cori Coarite, Jhon Alexander Chambi Vilca, Ángel Rosendo Condori-Coaquira, David Mamani-Pari, Milton Edward Humpiri-Flores, Esteban Tocto-Cano

**Affiliations:** Escuela Profesional de Ingeniería de Sistemas, Universidad Peruana Unión, Juliaca, Perú

**Keywords:** computer vision, deep learning, environmental robustness, parking space detection, smart parking systems, YOLO

## Abstract

**Introduction:**

Automated parking-space detection is an increasingly important component of intelligent urban mobility because inefficient parking searches contribute to travel delays, congestion, fuel consumption, and emissions.

**Methods:**

This systematic literature review, conducted using Kitchenham's methodology and the PICOC framework, synthesizes 15 peer-reviewed studies published between 2021 and March 2026. It examines detection methods, reported accuracy and computational efficiency, environmental evaluation coverage, dataset use, preprocessing practices, reproducibility, and future research directions.

**Results:**

YOLO-based architectures were the most frequently adopted model family, appearing in seven of the 15 studies (46.7%). Selected studies reported mean average precision values above 90% and processing speeds above 40 FPS; however, cross-study comparisons were limited by heterogeneous datasets, hardware configurations, metric definitions, and experimental conditions. Environmental evaluation was limited and uneven: fog was examined in one of 15 studies, occlusion in two, and snow in none. PKLot and CNRPark-EXT were the most frequently used public benchmarks, whereas the use of private and mixed datasets limited external validation when data or metadata were not shared.

**Discussion:**

This review provides an analytical performance matrix and a quantified environmental evaluation-coverage framework that identify recurring gaps in performance reporting, environmental validation, and reproducibility. The findings demonstrate the need for standardized comparative benchmarks, environmentally diverse datasets, and transparent reporting practices to enable robust assessment of real-world performance, robustness, and scalability.

## 1 Introduction

As cities become denser and private vehicle ownership continues to rise, the search for parking has become a visible source of urban inefficiency, adding avoidable travel time, intensifying congestion, and increasing fuel use and emissions. The pressure is especially pronounced around shopping centers, university campuses, and hospitals, where demand fluctuates sharply and parking shortages can spill over into surrounding road networks, increasing both traffic delays and operating costs. Empirical studies suggest that a non-trivial share of urban traffic consists of drivers circling for available spaces, a pattern that challenges the assumption that parking is merely a static land-use issue rather than an active contributor to congestion (Lu et al., 2025). This has led to recent research on computer vision and deep learning techniques that identify parking-space occupancy from real-time visual data. Compared with conventional physical sensors, these approaches reduce dependence on intrusive infrastructure; for example, inductive loop detectors require complex and costly installation, whereas camera-based systems can be deployed with greater adaptability across heterogeneous urban settings (Thabit et al., 2024). Taken together, the evidence suggests a shift from hardware-intensive parking monitoring to more scalable, vision-based systems that can support the real-time management of urban parking demand.

Traditional parking management systems that rely on physical sensors face persistent barriers to large-scale deployment. High installation and maintenance costs, limited scalability in complex environments such as underground parking facilities and densely built urban areas, and the lack of integrated services including real-time occupancy updates, mobile payment, and user notification mechanisms all reduce their practical viability (Alam et al., 2023). To address these shortcomings, researchers have increasingly adopted Internet of Things (IoT) technologies, which enable capabilities such as smart sensing, remote authentication, continuous occupancy monitoring, and digital payment services. Despite these advances, IoT-based parking systems remain constrained by high energy consumption, interoperability issues across heterogeneous devices, and dependence on additional communication and sensing infrastructure (Alam et al., 2023). For policymakers and urban planners, these constraints may increase the deployment and maintenance requirements and complicate city-wide implementation. Camera-based alternatives may reduce dependence on space-level physical sensors, particularly when existing surveillance infrastructure can be reused. However, their actual cost-effectiveness must be established through context-specific economic evaluation.

Data privacy considerations also merit further attention. Camera-based systems deployed in public parking lots must comply with applicable personal-data regulations (e.g., GDPR in the European context). Techniques such as on-device anonymization and edge inference, which process images locally without transmitting identifiable information, are increasingly being explored to reconcile detection accuracy with privacy requirements. These practical deployment barriers—privacy compliance, integration with legacy infrastructure, and maintenance costs—are as important as technical accuracy when evaluating the real-world viability of vision-based parking systems (Wong et al., 2023).

This systematic literature review examines the recent advances in automatic parking-space detection based on computer vision. Earlier reviews provided broader or earlier accounts of vision-based parking systems (de Almeida et al., 2022; Wong et al., 2023); however, they did not jointly examine recent architectural choices, metric-reporting completeness, dataset accessibility, computational context, and environmental evaluation coverage within the period from 2021 to March 2026. This period includes the growing adoption of newer YOLO variants, transformer-based detectors, segmentation networks, and edge-oriented implementations. Therefore, the present review extends the existing literature through a focused synthesis, an analytical performance matrix, and a descriptive cross-tabulation of model families and reported environmental conditions (Min et al., 2021; Bui and Suhr, 2021; Yu et al., 2020). The present review extends this framework in two respects: first, it is applied within a corpus temporally bounded between 2021 and March 2026, enabling a focused analysis of recent methodological and evaluation trends; second, it identifies descriptive patterns between architectural choice and environmental evaluation coverage. Beyond this classification framework, the review assesses reported approaches using reported accuracy and computational-efficiency metrics while examining the datasets that underpin current research, with particular attention to their limitations and potential sources of bias. Because the corpus is small and heterogeneous, the observed patterns are interpreted descriptively rather than as statistical correlations. The resulting analysis identifies current methodological gaps and outlines directions for future research toward more robust and deployable smart parking systems.

Unlike narrative reviews, this Systematic Literature Review follows the methodological framework proposed by Kitchenham and Brereton (2013), a protocol widely adopted for its rigor, transparency, and reproducibility in synthesizing scientific evidence. Kitchenham's methodology is particularly valuable in the rapidly evolving field of computer vision for parking detection, where the proliferation of incremental YOLO variants and task-specific datasets makes it easy to conflate genuine architectural breakthroughs with dataset-specific optimizations. The structured appraisal process applied here provides a systematic basis for distinguishing robust contributions from more circumstantial findings, a capability that less structured reviews cannot reliably offer. This approach provides a stronger basis for interpreting the current state of research and for defining priorities that can guide subsequent investigations in automatic parking space detection and smart parking systems (Kitchenham and Brereton, 2013). Accordingly, the methodological framework informed the following research questions:

**RQ1**. What computer vision tasks and model families have been applied to automatic parking-space detection?
**RQ2**. What accuracy and computational-efficiency levels have been reported, and under which datasets, hardware configurations, and experimental conditions?
**RQ3**. To what extent have the proposed methods been evaluated under variations in illumination, weather, occlusion, and camera configuration?
**RQ4**. What public, private, and mixed datasets have been used, and what limitations do they present for generalization and reproducibility?
**RQ5**. What methodological limitations and future research priorities emerge from the reviewed studies?
**RQ6**. What preprocessing and data-augmentation practices were used during training and inference?

To address these research questions, this review adopted a systematic process for identifying, selecting, and analyzing relevant studies, as described in Section 2. Section 3 presents the findings of the structured analysis of the selected literature. Section 4 critically interprets these results in light of the review objectives and the broader research landscape, including a dedicated discussion of the review's limitations. Section 5 concludes the paper by summarizing the main findings and outlining directions for future research.

## 2 Methodology

This study follows the systematic review methodology proposed by Kitchenham and Brereton (2013), which organizes the review process into planning, execution, and reporting phases. The review begins with a structured search strategy based on carefully selected keywords designed to capture the relevant literature. The retrieved studies were then screened using predefined inclusion and exclusion criteria to ensure that only publications aligned with the review objectives were retained, reducing the likelihood of introducing irrelevant evidence into the analysis. The final set of studies undergoes a detailed critical assessment focused on answering the predefined research questions, providing a transparent and reproducible basis for synthesizing of the available evidence.

### 2.1 Phase 1: review planning

The planning phase established the conceptual and methodological foundation of the review by defining the research questions (Section 1) and developing the review protocol using the Population, Intervention, Comparison, Outcome, and Context (PICOC) framework. During this stage, the inclusion, exclusion, and quality assessment criteria were defined to provide a consistent basis for study selection and critical evaluation. Together, these components promote a transparent and reproducible review process, reducing potential sources of bias and strengthening the credibility of synthesized evidence.

#### 2.1.1 Protocol design using PICOC

The scope of the review was defined using the Population, Intervention, Comparison, Outcomes, and Context (PICOC) framework, which provides a structured basis for developing the review protocol and maintaining methodological consistency. By explicitly specifying each component of the research strategy, the framework supports a focused, transparent literature search that is reproducible. Table 1 presents the PICOC elements adopted in this study and the corresponding criteria used to guide the identification and selection of relevant publications.

**Table 1:** PICOC elements used in planning.

**P**	Computer vision methods for detecting parking spaces.
**I**	Application of deep learning models for automatic detection (YOLO, U-Net, etc.).
**C**	Comparison between types of approaches (object detection, segmentation, and graphs) and between specific models such as YOLO, Faster R-CNN, Mask R-CNN, etc.
**O**	Accuracy, processing speed, and robustness to environmental variations.
**C**	Real or simulated urban environments using images or video of parking lots.

#### 2.1.2 Inclusion and exclusion criteria

Explicit inclusion and exclusion criteria were established to ensure that the selected studies were relevant to the review objectives and met consistent methodological standards. These criteria guided the screening process by retaining recent empirical research focused on the application of computer vision and deep learning techniques for parking space detection in real-world settings, while excluding studies that fell outside the defined scope. This selection strategy promotes a transparent and reproducible review process and ensures that the evidence synthesis is based on methodologically sound and contextually relevant publication.

Tables 2, 3 present the inclusion and exclusion criteria, respectively, applied in this study.

**Table 2:** Inclusion criteria applied in the review.

Code	Description
CI01	Articles published between 2021 and March 2026 and indexed or publicly available in the selected databases by the end of the search period.
CI02	Published as peer-reviewed journal articles and retrieved through Web of Science, ScienceDirect, or Dimensions.
CI03	Empirical or original studies with practical applications in automatic parking space detection.
CI04	Written in English.
CI05	Relevant to the fields of computer vision, artificial intelligence, engineering, or computer science.
CI06	Apply computer vision and deep learning techniques for automatic parking-space or parking-occupancy detection.
CI07	Are available in full text for review and analysis.
CI08	Use or develop real or simulated datasets related to parking (e.g., PKLot, PS 2.0, SNU, etc.).

**Table 3:** Exclusion criteria applied in the review.

Code	Description
CE01	Articles published before 2021 or after March 2026.
CE02	Not published in indexed journals or without peer review.
CE03	Theoretical studies, narrative reviews, simulations without practical validation, or those that do not directly address parking space detection.
CE04	Written in languages other than English.
CE05	Related to fields outside of computer vision or artificial intelligence.
CE06	Do not apply image-based computer vision or deep learning methods to automatic parking-space or parking-occupancy detection.
CE07	The full text of the article is not available for consultation.
CE08	Do not use datasets related to parking or do not clearly specify their data source.

#### 2.1.3 Keywords and synonyms

The search strategy was developed by identifying the principal concepts derived from the research objectives and expanding them with relevant synonyms. These terms were systematically combined using Boolean operators to construct the search strings applied across the selected databases, increasing the retrieval coverage while reducing the likelihood of overlooking relevant studies because of differences in terminology. Table 4 summarizes the resulting search vocabulary, organized by conceptual category, and provides the basis for the literature retrieval process.

**Table 4:** Keywords and synonyms used in the search.

Keyword	Synonyms
Parking space detection	Car parking detection, parking spot recognition
Computer vision	Vision-based, visual analysis, and image processing
Deep learning	Neural networks, CNN, YOLO, R-CNN, and AI-based detection
Object detection	Target recognition, object localization
Semantic segmentation	Image segmentation, region-based analysis
Smart parking system	Intelligent parking, Automated parking management
Datasets	Public datasets, benchmark datasets, and annotated images
Model evaluation	Performance metrics, accuracy measurement, and Benchmarking
Robustness	Generalization, Stability, Cross-dataset, and performance
Real-world conditions	Environmental variation, Illumination, Weather, and Camera angle

The temporal restriction defined in criteria CI01 and CE01 (2021–March 2026) applies exclusively to the primary studies included in this evidence synthesis. Foundational methodological references, such as the systematic review guidelines proposed by Kitchenham and Brereton, together with a limited number of background studies cited in the Introduction to contextualize the research field, are not subject to this restriction because they are cited for methodological or contextual purposes rather than being included in the evidence synthesis.

### 2.2 Phase 2: Conducting the review

#### 2.2.1 Information sources and search syntax

A core Boolean search strategy was developed from predefined concepts and adapted separately to the syntax and searchable fields of each database. Conceptual equivalence was maintained across databases, whereas operators, field codes, filters, and quotation conventions were adapted to each platform. The literature search was conducted in Web of Science, ScienceDirect, and Dimensions and was updated until March 2026.

Web of Science, ScienceDirect, and Dimensions were selected because they provide complementary coverage of peer-reviewed research in engineering, artificial intelligence, and computer vision. IEEE Xplore was not searched as a standalone source. Consequently, IEEE-exclusive conference papers and records not indexed in the selected sources may have been overlooked. Because the eligibility criteria prioritized peer-reviewed journal articles, the resulting synthesis should be interpreted as primarily journal-focused rather than an exhaustive representation of all conference and journal research. Future updates should include a targeted IEEE Xplore search to evaluate the effect of this omission. This potential limitation is discussed further in Section 4.

The following syntax was used:

(“parking detection” OR “smart parking” OR “parking space detection”) AND (“computer vision” OR YOLO OR “deep learning”) AND (dataset OR “real-world conditions”) AND (evaluation OR robustness)

#### 2.2.2 Study assessment and selection

Following the retrieval of the search results, the study selection process was conducted in three sequential stages: title, abstract, and full-text screening. At each stage, the predefined inclusion and exclusion criteria were applied to refine the set of candidate studies and exclude publications that were outside the scope of the review. The studies retained after the full-text eligibility assessment were evaluated using a seven-criterion methodological quality rubric, CC01–CC07, as detailed in Table 5. This appraisal provides a consistent basis for evaluating the reliability of the evidence and ensures that the final synthesis is informed by studies that meet the methodological standards established for this review.

**Table 5:** Quality criteria.

Code	Quality criterion
CC01	Does the study present clear research objectives and justification?
CC02	Are methods, models, or architectures described in detail, including key hyperparameters and training procedures?
CC03	Are quantitative results reported using standard metrics (mAP, IoU, FPS, and latency)?
CC04	Are limitations discussed and future directions proposed?
CC05	Is a public dataset used or the dataset source clearly described?
CC06	Are results experimentally validated or compared with other methods?
CC07	Are sufficient implementation details provided for reproducibility, including hardware, software framework, and availability of code or model weights?
**Score:** Yes = 2 Partially = 1 No = 0 Total range = 0–14 Inclusion threshold ≥ 8	

#### 2.2.3 Data extraction and coding

To address the predefined research questions consistently, a structured data extraction framework was developed, with specific extraction categories defined for each question. This framework standardizes the collection of relevant information across the selected studies and supports both comparative and thematic analyses. Table 6 summarizes the extraction categories associated with each research question, providing a transparent link between the review objectives and the evidence synthesized from primary studies.

**Table 6:** Categories of data extracted by research question.

Research question	Extraction categories
What computer vision methods have been used to detect parking spaces and how are they grouped (object detection, segmentation, and graphs)?	Methods, Models, and Grouping
What level of accuracy (mAP, IoU) and processing speed (FPS, latency) does the proposed method achieve according to experimental results?	Model, Accuracy, and Speed
What impact do environmental variations (lighting, precipitation) and capture parameters have on the performance of detection methods?	Environment, Performance, Methods, and Observation
What datasets (public and private) are used to train and evaluate the methods, and what are their main characteristics and limitations?	Datasets, Accessibility, Characteristics, and Limitations
What improvements or future research directions do the authors propose to overcome the limitations of their study?	Proposals, Future, and Improvement
What preprocessing techniques (such as filtering, color transformation, or data augmentation) have been used in the reviewed studies, and how are they classified according to their role in the automatic detection process?	Preprocessing used, Type, Role, and Model

#### 2.2.4 Study selection process

The study selection process comprised three sequential stages: title screening, abstract screening, and full-text assessment. During each stage, the inclusion and exclusion criteria established in Phase 1 were applied to refine the candidate study pool. This staged screening procedure improved the consistency of the selection process and helped ensure that only studies meeting the predefined review scope were retained for subsequent phases.

**Figure 1:**
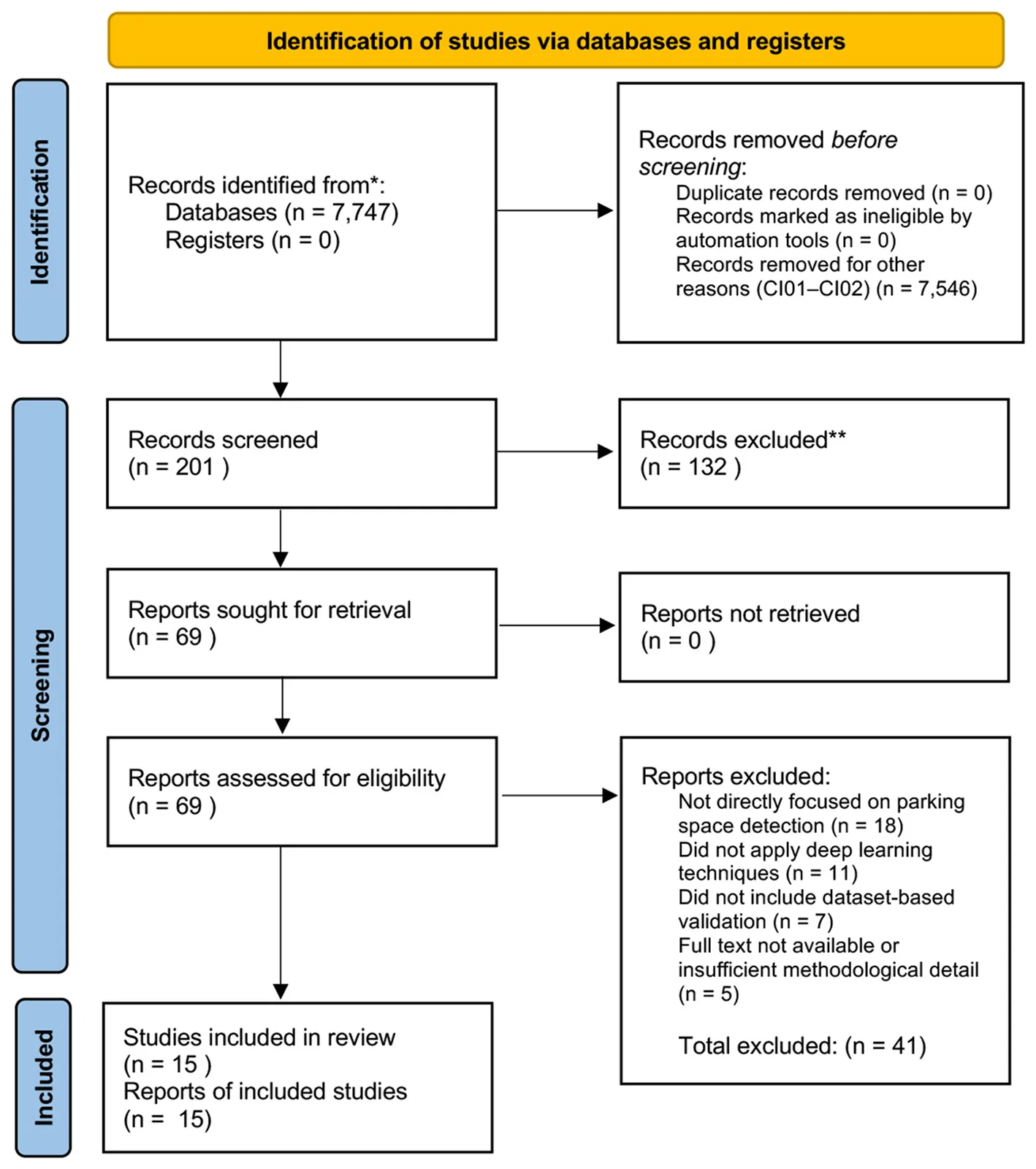
Search and study selection flow diagram. Exclusion counts are reported at two distinct stages: **(i)** full-text assessment against inclusion/exclusion criteria CE01–CE08 (*n* = 41), and **(ii)** quality appraisal against the revised seven-criterion rubric (Table 5; *n* = 13). These two stages are sequential, not additive.

Figure 1 presents the identification, screening, full-text eligibility, quality-appraisal, and inclusion stages. The diagram disaggregates the results by database, providing transparency regarding the contribution of each source to the final study set.

The study-selection process is summarized as follows. The initial search across the three databases retrieved 7,747 records. After applying the publication year and subject area filters (CI01–CI02), 201 studies remained for screening. Abstract screening reduced this number to 69 studies, which were subsequently assessed in the full text. Twenty-eight articles satisfied the full-text eligibility criteria (CE01–CE08). These articles were then evaluated using a revised seven-criterion methodological quality rubric. Thirteen studies scoring below 8 of the maximum 14 points were excluded, leaving 15 studies in the final synthesis. The 41 full-text exclusions and 13 quality-appraisal exclusions corresponded to sequential and distinct stages and should therefore be reported separately.

#### 2.2.5 Study selection and bias control

The review protocol was not prospectively registered in a public repository. The research questions, search strategy, eligibility criteria, and original six-criterion quality rubric were established prior to screening. During the revision, CC07 was added as a documented methodological amendment to assess implementation reproducibility. Several measures were implemented to mitigate the potential selection bias. First, study screening and evaluation were conducted according to the predefined criteria reported in Tables 2, 3, respectively, together with the revised seven-criterion quality-assessment rubric in Table 5. Second, the full-text eligibility and quality-assessment decisions were independently reviewed by two co-authors, and any disagreements were resolved through consensus, following the underlying principle of independent assessment recommended in PRISMA 2020. Third, the search strategy was applied consistently across the selected databases, with only the restrictions specified in CI01 and CI04, while the database selection was based on complementary disciplinary coverage. These procedures do not eliminate all possible sources of bias, but they provide a transparent and reproducible basis for controlling their influence within the scope of this review.

Potential duplicates were identified using normalized DOI, title, and author fields, followed by a manual inspection of possible near-duplicate records. No exact duplicates were identified under this procedure (*n* = 0); however, records with incomplete or inconsistent metadata may not have been detected as duplicate records. Studies based on shared public datasets, such as PKLot or CNRPark-EXT, were retained when they introduced distinct models, methodologies, or experimental settings. The reuse of a dataset was therefore not considered sufficient evidence of duplication, as different studies may address separate research objectives or propose independent technical contributions.

During the full-text assessment, five studies were excluded because they did not provide sufficient methodological information for reliable quality evaluation. The most frequent issues were the absence of reported performance metrics (e.g., mAP, IoU, or FPS; criterion CC03) and insufficient descriptions of the datasets employed (criterion CC05). The exclusion of these studies may have affected the observed distribution of methods and evaluation scenarios; however, the direction and magnitude of this effect could not be determined from the available information. The retained studies showed recurring patterns, but these patterns could not establish that the excluded studies would have produced equivalent findings. Therefore, this uncertainty is treated as a limitation of the quality-based selection procedure and the transparency of the primary literature.

## 3 Results

This section reports the findings obtained from the analysis of the 15 scientific articles selected for this review, focusing on automatic parking space detection through computer vision and artificial intelligence techniques. The evidence is organized around six research questions (RQs), with comparative information summarized in tables and complemented by a contextual discussion of the reviewed studies.

### 3.1 RQ1: Computer vision tasks and model families used for parking-space detection

#### 3.1.1 Classification note

Park (2021) used BLE beacon signals and a shallow 1D-CNN instead of image-based computer vision. Therefore, it is treated as a complementary non-visual approach and is not interpreted as evidence of the prevalence or performance of image-based architectures.

Table 7 summarizes the distribution of computer vision techniques. Bounding box-based detection dominates, appearing in seven studies (Duong et al., 2023; Balusamy et al., 2024; Aswath et al., 2025; Tang et al., 2024; Morell et al., 2025; Prova et al., 2022; Zhang et al., 2024), reflecting the maturity of detectors such as YOLO in real-time scenarios. Image classification (Farley et al., 2021; Kaur et al., 2021) and hybrid bounding-box plus time-series approaches (Nguyen and Sartipi, 2025; Xu et al., 2024) address the temporal occupancy dynamics. Graph neural networks (GNNs) and active segmentation remain less frequent (Lu et al., 2025; Grbić and Koch, 2023), indicating ongoing exploration rather than consolidation of these methods. Park (2021) used BLE beacon signals rather than image-based detection and was classified as a non-visual approach.

**Table 7:** Distribution of detection and supporting techniques used in the reviewed studies.

Method	Source
Image classification	Farley et al., 2021; Kaur et al., 2021
Bounding box-based detection	Duong et al., 2023; Balusamy et al., 2024; Aswath et al., 2025; Tang et al., 2024; Morell et al., 2025; Prova et al., 2022; Zhang et al., 2024; Rafique et al., 2023
Bounding box-based detection + time series prediction	Nguyen and Sartipi, 2025; Xu et al., 2024
Bounding box detection, semantic segmentation, direct regression, and localization with GNN	Grbić and Koch, 2023
Semantic segmentation + active deep learning	Lu et al., 2025
Non-visual BLE signal processing with shallow 1D-CNN	Park, 2021

Table 8 summarizes the deep neural network architectures adopted in the analyzed studies. This classification provides a view of the architectural choices underlying current computer vision-based parking systems, revealing which model families have achieved greater adoption and which remain unexplored. The observed distribution helps characterize the maturity of different deep learning approaches within this application domain and establishes a basis for comparing their prevalence across the reviewed literature.

**Table 8:** Distribution of neural network models used in the analyzed studies.

Neural network model	Studies that use it
YOLO	Balusamy et al., 2024; Aswath et al., 2025; Tang et al., 2024; Morell et al., 2025; Grbić and Koch, 2023; Zhang et al., 2024; Rafique et al., 2023
CNN	Kaur et al., 2021; Xu et al., 2024; Park, 2021; Prova et al., 2022
Classic models (LeNet, AlexNet)	Farley et al., 2021
Deformable DETR	Nguyen and Sartipi, 2025
OcpDet (occupancy detector)	Duong et al., 2023
U-Net	Lu et al., 2025

The reviewed studies show a marked preference for *YOLO*-based architectures, including variants from YOLOv4 to YOLOv8—with versions v5 and v8 appearing most frequently appearing in seven articles (Balusamy et al., 2024; Aswath et al., 2025; Tang et al., 2024; Morell et al., 2025; Grbić and Koch, 2023; Zhang et al., 2024; Rafique et al., 2023). Their widespread adoption is associated with the balance these detectors provide between computational efficiency and detection performance, making them suitable for real-time parking applications. In comparison, conventional CNN architectures, including LeNet and AlexNet, have been mainly reported in studies focusing on simpler classification scenarios (Farley et al., 2021; Kaur et al., 2021; Xu et al., 2024; Park, 2021). Other studies have explored more specialized architectures, such as the custom *OcpDet* model (Duong et al., 2023) and transformer-based *Deformable DETR* approach (Nguyen and Sartipi, 2025), which target more demanding detection settings. The use of *U-Net* (Lu et al., 2025) further illustrates the relevance of segmentation-based methods when pixel-level representations of parking environments are required, particularly in decentralized deployment scenarios.

Table 9 summarizes the distribution of the selected articles according to the primary computer vision tasks addressed. Object detection is the dominant task, appearing in 10 studies (Duong et al., 2023; Balusamy et al., 2024; Nguyen and Sartipi, 2025; Aswath et al., 2025; Tang et al., 2024; Morell et al., 2025; Prova et al., 2022; Grbić and Koch, 2023; Zhang et al., 2024; Rafique et al., 2023). This predominance reflects the practical requirements of parking management systems, where vehicle locations must be identified and slot occupancy must be determined under real-time constraints.

**Table 9:** Distribution of articles by computer vision task.

Group/category	Sources
Object detection	Duong et al., 2023; Balusamy et al., 2024; Nguyen and Sartipi, 2025; Aswath et al., 2025; Tang et al., 2024; Morell et al., 2025; Prova et al., 2022; Grbić and Koch, 2023; Zhang et al., 2024; Rafique et al., 2023
Image segmentation	Lu et al., 2025; Grbić and Koch, 2023
Image classification	Farley et al., 2021
Complementary system components (license-plate recognition, temporal occupancy prediction, and non-visual sensing)	Kaur et al., 2021; Xu et al., 2024; Park, 2021

Segmentation-based approaches appear less frequently, with two studies exploring this task (Lu et al., 2025; Grbić and Koch, 2023) to obtain more detailed spatial representations of parking scenes. Image classification is the least represented category, with only one study (Farley et al., 2021) adopting a CNN-based strategy to determine parking-space availability. This distribution suggests a shift from simpler image-level recognition methods to detection-oriented solutions capable of supporting more complex and dynamic parking scenarios.

Three additional studies (Kaur et al., 2021; Xu et al., 2024; Park, 2021) investigate complementary components of smart parking systems beyond the primary computer vision tasks analyzed in the previous categories. These studies addressed license plate recognition, time-series prediction using long short-term memory (LSTM) networks, and Internet of Things (IoT)-based parking management approaches. Their presence in the reviewed literature illustrates a broader trend toward integrated parking solutions, where visual perception is combined with temporal analysis and connected infrastructures to support more comprehensive system functionalities.

To examine the extent of YOLO predominance without overstating its role, the distribution of architectures across the 15 studies was quantified in this study. YOLO-based detectors represent the most frequently adopted architectural family, appearing in seven studies (46.7%). However, this frequency does not constitute an absolute majority, as the remaining eight studies (53.3%) relied on a diverse range of alternatives, including custom detectors (OcpDet), transformer-based models (Deformable DETR), segmentation networks (U-Net), classical convolutional architectures (LeNet, AlexNet, and CaffeNet), hybrid temporal models (CNN–LSTM), and sensor-oriented approaches (1D-CNN). This distribution suggests that YOLO has become the most common individual choice owing to its practical balance between accuracy and inference speed; however, the field has not converged on a single dominant architecture. Instead, model selection appears to remain dependent on the specific task, data characteristics, and deployment requirements of each study. The YOLO variants represented in the corpus range from YOLOv4 to YOLOv8, which differ considerably in terms of architecture and computational complexity; therefore, they should not be interpreted as a fully homogeneous category. In addition, none of the reviewed studies performed a direct comparison between YOLO-based models and alternatives such as Deformable DETR, U-Net, or graph-based approaches under equivalent experimental conditions. Consequently, the available evidence supports the conclusion that YOLO is the most *widely adopted* family in this domain, but does not demonstrate its universal superiority over other architectures. Observed adoption patterns may also be influenced by factors such as open-source availability, implementation maturity, and research community adoption, rather than only accuracy improvements.

The distinction between traditional and deep learning-based methods was defined using two complementary criteria applied during the screening and data-extraction phases. A method was categorized as *traditional* when its operation depended primarily on manually designed features, such as color histograms, edge-based descriptors (e.g., Canny or Hough transforms), morphological operations, or background subtraction, without including trainable deep neural-network components. In contrast, methods were classified as *deep learning-based* when they incorporated at least one end-to-end trainable neural-network component for feature representation, detection, or classification, even when additional shallow processing stages, such as SVM classifiers, were applied afterwards. Approaches that combined both strategies were assigned to the *hybrid* category.

This classification framework was applied during data extraction (see Table 6) and cross-checked against the quality criterion CC02, which required a detailed description of the employed architecture (Table 5). Under this classification, only one study, Park (2021) study was identified as primarily traditional. This study relies on BLE beacon signals processed through a shallow neural network rather than image-based deep learning. The remaining 14 studies employed image-based deep learning approaches, indicating that, within the reviewed corpus, recent research has been strongly oriented toward trainable visual models rather than hand-crafted feature pipelines during the 2021–2025 period.

### 3.2 RQ2: Reported accuracy and computational efficiency across datasets and hardware platforms

Table 10 summarizes the deep neural network models adopted in the reviewed studies. YOLO variants (v4, v5, v8, and modified versions incorporating mechanisms such as CBAM) represent the most frequent architectural choice, appearing in several articles (Balusamy et al., 2024; Aswath et al., 2025; Tang et al., 2024; Morell et al., 2025; Grbić and Koch, 2023; Zhang et al., 2024; Rafique et al., 2023). Their prevalence is associated with the practical balance they provide between detection performance and computational efficiency, which is particularly relevant to real-time parking applications.

**Table 10:** Models used in the analyzed articles.

Model	Articles
OcpDet	Duong et al., 2023
YOLOv5	Balusamy et al., 2024; Grbić and Koch, 2023; Zhang et al., 2024; Rafique et al., 2023
YOLOv8	Aswath et al., 2025; Morell et al., 2025; Zhang et al., 2024
YOLOv4 + CBAM	Tang et al., 2024
YOLOv7	Zhang et al., 2024
YOLOv5n/v5x/v8n/v8x	Zhang et al., 2024
Faster R-CNN	Zhang et al., 2024
SSD MobileNetV2	Zhang et al., 2024
Mask R-CNN	Grbić and Koch, 2023
ResNet-18	Grbić and Koch, 2023
GCN-based model	Grbić and Koch, 2023
Deformable DETR	Nguyen and Sartipi, 2025
U-Net	Lu et al., 2025
Inception V3 + BAT Opt.	Lu et al., 2025
LeNet/AlexNet/mLeNet	Farley et al., 2021
CNN	Kaur et al., 2021; Xu et al., 2024
CNN + LSTM	Xu et al., 2024
1D-CNN	Park, 2021
CaffeNet	Prova et al., 2022

The reviewed literature also includes alternative architectures, such as *Mask R-CNN, U-Net*, and *Deformable DETR*, which are adopted in scenarios where a more detailed spatial representation or advanced detection capabilities are required. Alongside these models, hybrid and classical architectures, including *CNN+LSTM, CaffeNet*, and *Inception V3*, illustrate the methodological diversity of the field by addressing complementary tasks such as temporal prediction, image classification, and object recognition.

Table 11 summarizes the reported precision-related results across the analyzed studies, primarily based on metrics such as mean average precision (mAP). Most studies that report this measure achieve values above 90%, with particularly high results observed by (Duong et al., 2023) and (Rafique et al., 2023), who reported mAP values of 99% and 99.5%, respectively. These results suggest a strong detection performance under the experimental conditions defined by each study. However, direct comparison remains limited because the evaluated datasets, experimental protocols, and metric configurations are not uniform across the literature. In addition, four of the 15 studies (26.7%) did not report mAP or an equivalent precision-related measure, restricting a comprehensive assessment of model performance across the reviewed corpus.

**Table 11:** Accuracy achieved by the models proposed in the analyzed articles.

Accuracy	Articles
mAP = 99%	Duong et al., 2023
mAP = 99.5%	Rafique et al., 2023
mAP = 98.13%	Kaur et al., 2021
mAP = 97%	Balusamy et al., 2024; Xu et al., 2024
mAP = 93.15%	Farley et al., 2021
mAP = 92.7%	Nguyen and Sartipi, 2025
mAP = 90%	Grbić and Koch, 2023
mAP = 87%	Zhang et al., 2024
mAP = 79.7%	Aswath et al., 2025; Lu et al., 2025
Not reported (N/R)	Tang et al., 2024; Park, 2021; Morell et al., 2025; Prova et al., 2022

Table 12 summarizes the reported processing speed of the evaluated models, which are primarily expressed in frames per second (FPS). Several studies achieve processing rates above 40 FPS (Balusamy et al., 2024; Grbić and Koch, 2023; Rafique et al., 2023), indicating potential for real-time inference under the specific hardware and experimental conditions reported. In contrast, lower processing rates have been reported in some studies, such as Farley et al. (2021), which achieves 2 FPS and may be insufficient for applications requiring continuous and low-latency monitoring. However, a valid direct comparison is not possible without standardized datasets, hardware, input resolution, batch size, and metric definitions. Moreover, eight of the 15 studies (53.3%) did not report processing speed, limiting the assessment of computational efficiency in the reviewed approaches.

**Table 12:** Processing speed achieved by the proposed models.

Speed	Articles
52 FPS	Balusamy et al., 2024
45 FPS	Rafique et al., 2023
≈45 FPS	Grbić and Koch, 2023
16.4 FPS	Zhang et al., 2024
15 to 20 FPS	Lu et al., 2025
8–15 FPS	Aswath et al., 2025
2 FPS	Farley et al., 2021
Not reported (N/R)	Duong et al., 2023; Nguyen and Sartipi, 2025; Kaur et al., 2021; Xu et al., 2024; Tang et al., 2024; Park, 2021; Morell et al., 2025; Prova et al., 2022

To provide a contextualized comparison across studies, Table 13 summarizes the main characteristics of the 15 reviewed studies, including the model, dataset scale, reported hardware, accuracy metrics, processing speed, and evaluated environmental conditions. This analysis highlights two recurring limitations: hardware specifications are reported only in a subset of studies, restricting comparisons between accuracy and computational efficiency, and dataset scales vary substantially (from 160 to over 695,000 annotated instances), limiting the interpretation of the reported accuracy values. Moreover, eight out of 15 studies (53%) omit processing speed and four out of 15 (27%) omit mAP, indicating that incomplete metric reporting remains a relevant challenge for cross-study evaluation.

**Table 13:** Analytical performance matrix: model, dataset, dataset scale, hardware, accuracy, speed, and environmental conditions.

Study	Model	Dataset	Dataset scale	Hardware	mAP	Speed	Conditions evaluated
1	OcpDet	PKLot + SNU-SPS	3,500 img (SNU-SPS)	N/R	99%	N/R	Sunny, rain, and cloudy
2	Custom YOLOv5 + FPN + ResNet	Google Open Images	N/R (subset)	Low-compute (N/R)	97%	52 FPS	N/R
3	Deformable DETR	PKLot, CityFlow, VeRi-776	12,000+ img (PKLot)	N/R	92.7%	N/R	Sunny, rain, and cloudy
4	YOLOv8 + Tesseract	Own (campus)	Small (N/R)	Raspberry Pi 4B	79.7%	8–15 FPS	Rain, night, and shadows
5	U-Net + Inception V3 + BAT	PKLot	12,417 img; 695,900 lbl	Server-based	79.7%	15–20 FPS	Rain, night, and shadows
6	LeNet / AlexNet / mLeNet	CNRPark-EXT + own	144,965 img	N/R	93.15%	2 FPS	Sunny, cloudy, and rain
7	CNN (ALPR)	Own	160 img	N/R	98.13%	N/R	Rain, night, shadows, and fog
8	CNN + LSTM	Own (62 days)	1,158 records	N/R	97%	N/R	Sunny, shadows, and night
9	YOLOv4 + CBAM	Own (campus)	N/R	N/R	N/R	N/R	Day, night, and shadows
10	1D-CNN (BLE)	Own (BLE)	425 samples	Smartphone + BLE	N/R	N/R	Shadows, lighting
11	YOLOv8x + Detectron2	Own (89 Málaga cams)	89 cameras	N/R	N/R	61 FPS (v8n); 13 FPS (D2)	Day/night, varied climate
12	CaffeNet	PKLot + CNRPark-EXT	400,000+ img	Edge devices	N/R	N/R	Occlusions, shadows, and light
13	YOLOv5x + Mask R-CNN + GCN	PKLot + CNRPark-EXT	695k + 144k lbl	N/R	90%	≈45 FPS	Sunny, cloudy, and rain
14	YOLOv5/v7/v8 + Faster R-CNN + SSD	Own (Roboflow)	377 img	N/R	87%	16.4 FPS	Lighting, occlusions
15	YOLOv5s	PKLot + MS COCO + own	PKLot-scale	IP cameras	99.5%	45 FPS	Sunny, shadows (day/night)

N/R indicates that the corresponding study did not report the information. Missing hardware specifications prevent rigorous interpretation of computational efficiency and edge-deployment feasibility. Dataset scales, annotation units, class distributions, and train–test partitions vary substantially across studies, limiting the direct interpretation of reported accuracy values.

### 3.3 RQ3: Environmental and image-capture conditions considered in model evaluation

Table 14 summarizes the environmental and image-capture conditions reported in the evaluation of the reviewed methods. Most studies examine sunny and rainy scenarios (Duong et al., 2023; Nguyen and Sartipi, 2025; Aswath et al., 2025; Farley et al., 2021; Grbić and Koch, 2023; Rafique et al., 2023), reflecting a primary focus on common outdoor conditions. Several studies have also incorporate challenging illumination factors, including night-time operation, shadows, and light variations (Aswath et al., 2025; Lu et al., 2025; Kaur et al., 2021; Xu et al., 2024; Tang et al., 2024; Prova et al., 2022). In contrast, more complex conditions, such as fog and occlusions, have received limited attention, suggesting that current evaluations still provide incomplete evidence of model robustness under diverse real-world scenarios.

**Table 14:** Environmental conditions in the analyzed studies.

Condition	Sources
Sunny	Duong et al., 2023; Nguyen and Sartipi, 2025; Farley et al., 2021; Xu et al., 2024; Grbić and Koch, 2023; Rafique et al., 2023
Rain	Duong et al., 2023; Nguyen and Sartipi, 2025; Aswath et al., 2025; Lu et al., 2025; Farley et al., 2021; Kaur et al., 2021; Morell et al., 2025
Cloudy	Duong et al., 2023; Nguyen and Sartipi, 2025; Farley et al., 2021; Grbić and Koch, 2023
Night	Aswath et al., 2025; Lu et al., 2025; Kaur et al., 2021; Xu et al., 2024; Tang et al., 2024; Morell et al., 2025
Shadows	Aswath et al., 2025; Lu et al., 2025; Kaur et al., 2021; Xu et al., 2024; Tang et al., 2024; Park, 2021, Morell et al., 2025; Prova et al., 2022; Rafique et al., 2023
Lighting	Park, 2021; Morell et al., 2025; Zhang et al., 2024
Occlusions	Prova et al., 2022; Zhang et al., 2024
Fog	Kaur et al., 2021
Snow	None
N/R	Balusamy et al., 2024

Table 15 quantifies the reported environmental evaluation coverage. Shadows (60%) and rain (47%) were addressed in approximate half of the studies, whereas fog (6.7%) and occlusions (13.3%) were markedly underrepresented. Snow was not evaluated in the reviewed corpus, representing an evidence gap for regions where snowy conditions are operationally relevant. The mean number of adverse conditions evaluated per study was 1.7 (SD = 1.2). No study evaluated all major adverse conditions considered in this review—rain, night-time operation, shadows, lighting variation, fog, occlusion, and snow—within a common experimental protocol, and six studies assess fewer than two such conditions.

**Table 15:** Quantitative coverage of environmental conditions.

Condition	Studies (*n*)	Coverage (%)
Shadows	9	60.0
Rain	7	46.7
Sunny	6	40.0
Night	6	40.0
Cloudy	4	26.7
Lighting variations	3	20.0
Occlusions	2	13.3
Fog	1	6.7
Snow	0	0.0

Table 16 summarizes the performance metrics reported in the analyzed studies for evaluating the model accuracy and efficiency. Precision is the most frequently used metric, appearing in nine studies (Duong et al., 2023; Aswath et al., 2025; Lu et al., 2025; Farley et al., 2021; Kaur et al., 2021; Xu et al., 2024; Morell et al., 2025; Grbić and Koch, 2023; Zhang et al., 2024), followed by recall (Duong et al., 2023; Aswath et al., 2025; Lu et al., 2025; Kaur et al., 2021; Morell et al., 2025; Grbić and Koch, 2023; Zhang et al., 2024) and F1-score (Aswath et al., 2025; Lu et al., 2025; Zhang et al., 2024). Together, these metrics provide a conventional basis for assessing the ability of the proposed method to correctly identify parking occupancy states.

**Table 16:** Performance metrics used in the analyzed studies.

Performance metric	Sources
mAP (mean Average Precision)	Duong et al., 2023
mAP	Balusamy et al., 2024; Morell et al., 2025
mAP@0.5	Aswath et al., 2025; Zhang et al., 2024
IoU (Intersection over Union)	Duong et al., 2023; Park, 2021
AR75, mAR40_90, AP50	Nguyen and Sartipi, 2025
F1-scores—confidence / precision—confidence	Balusamy et al., 2024
Precision	Duong et al., 2023; Aswath et al., 2025; Lu et al., 2025; Farley et al., 2021; Kaur et al., 2021; Xu et al., 2024, Morell et al., 2025; Grbić and Koch, 2023; Zhang et al., 2024
Recall	Duong et al., 2023; Aswath et al., 2025; Lu et al., 2025; Kaur et al., 2021; Morell et al., 2025; Grbić and Koch, 2023, Zhang et al., 2024
F1-score	Aswath et al., 2025; Lu et al., 2025; Zhang et al., 2024
Sensitivity/Specificity / FPR / FNR	Lu et al., 2025
Recognition Rate	Kaur et al., 2021
MAE, RMSE	Xu et al., 2024
Accuracy (Acc)	Park, 2021
FPS (real-time)	Morell et al., 2025
High accuracy / robustness / real-time	Rafique et al., 2023
Processing time	Farley et al., 2021
Not reported (N/R)	Tang et al., 2024; Prova et al., 2022

Object-detection-oriented metrics, particularly mean average precision (*mAP*) and related variants such as *mAP@0.5, AR75*, and *AP50*, are also widely adopted (Duong et al., 2023; Balusamy et al., 2024; Aswath et al., 2025; Tang et al., 2024; Morell et al., 2025; Grbić and Koch, 2023; Zhang et al., 2024), which is consistent with the predominance of detection-based approaches in the reviewed corpus. Other studies employ complementary measures, including Intersection over Union (IoU), mean absolute error (MAE), root mean square error (RMSE), and confidence curves, to evaluate additional aspects such as localization accuracy or prediction error. Nevertheless, two studies (Tang et al., 2024; Prova et al., 2022) do not report evaluation metrics, limiting the transparency and direct comparability of their results.

Table 17 summarizes the main robustness-related aspects reported in the studies analyzed. Most articles focus on the ability of the proposed methods to cope with challenging environmental factors, including weather changes, illumination variability, shadows, and occlusions, which are recurrent obstacles in practical parking scenarios. Other studies have addressed more specific robustness dimensions, such as mitigating the impact of imbalanced datasets (Balusamy et al., 2024), reducing reliance on manual annotations (Grbić and Koch, 2023), improving tolerance to illumination changes through advanced architectures (Zhang et al., 2024), and maintaining stable real-time performance under varying operating conditions (Rafique et al., 2023).

**Table 17:** Robustness aspects addressed by the analyzed articles.

Observation	Sources
Robustness to environmental conditions (weather, lighting, shadows, occlusions)	Duong et al., 2023; Nguyen and Sartipi, 2025; Aswath et al., 2025; Lu et al., 2025; Farley et al., 2021; Kaur et al., 2021; Xu et al., 2024; Tang et al., 2024; Park, 2021; Morell et al., 2025; Prova et al., 2022; Grbić and Koch, 2023
Robustness against imbalanced data or noise in the dataset	Balusamy et al., 2024
Robustness without the need for manual labeling / improved system efficiency	Grbić and Koch, 2023
Robustness in advanced models (YOLOv8x, YOLOv8n) against variable lighting and occlusions	Zhang et al., 2024
Robustness in overall performance (real-time, accuracy, FPS, etc.)	Rafique et al., 2023

### 3.4 RQ4: Dataset accessibility, representativeness, and reproducibility limitations

Table 18 summarizes the datasets used to train and evaluate the proposed methods. Public benchmarks such as PKLot and CNRPark-EXT are frequently adopted (Lu et al., 2025; Farley et al., 2021; Prova et al., 2022; Grbić and Koch, 2023), reflecting their continued use in parking occupancy detection research. Some studies combine multiple data sources to increase data diversity and improve model generalization (Zhang et al., 2024), while others rely on custom datasets (Aswath et al., 2025; Kaur et al., 2021; Xu et al., 2024; Tang et al., 2024; Park, 2021; Morell et al., 2025; Rafique et al., 2023) designed for specific environmental and operational conditions. Large-scale general-purpose datasets, such as ImageNet (Duong et al., 2023) and Open Images (Balusamy et al., 2024), are also used for pre-training, providing broader visual representations that support transfer learning in parking-related tasks.

**Table 18:** Datasets used in the analyzed articles.

Dataset	Sources
ImageNet	Duong et al., 2023
Google Open Images Dataset	Balusamy et al., 2024
PKLot + SNU-SPS	Duong et al., 2023
PKLot	Lu et al., 2025
CNRPark-EXT + own	Farley et al., 2021
PKLot and CNRPark-EXT	Morell et al., 2025; Prova et al., 2022
PKLot, CNRPark-EXT, and own	Grbić and Koch, 2023
PKLot, MS COCO, and own	Zhang et al., 2024
PKLot, CityFlow, VeRi-776	Nguyen and Sartipi, 2025
Own dataset	Aswath et al., 2025; Kaur et al., 2021; Xu et al., 2024; Tang et al., 2024; Park, 2021; Rafique et al., 2023

Table 19 presents the accessibility of the datasets used in the studies, classified into public-only, private-only, and mixed (combining public benchmarks with proprietary data) categories. Studies using exclusively public datasets facilitate reproducibility and direct comparison across methods. Studies relying on private or mixed datasets may constrain external validation when data or metadata are not shared.

**Table 19:** Accessibility of the datasets used.

Accessibility	Sources
Public	Duong et al., 2023; Balusamy et al., 2024; Nguyen and Sartipi, 2025; Lu et al., 2025; Farley et al., 2021; Morell et al., 2025, Prova et al., 2022; Grbić and Koch, 2023; Zhang et al., 2024; Rafique et al., 2023
Private	Aswath et al., 2025; Kaur et al., 2021; Xu et al., 2024; Tang et al., 2024; Park, 2021

Table 20 summarizes the main characteristics of the datasets used across the analyzed studies. The reviewed datasets exhibit substantial variation in both design and intended application. Some studies focus on scenarios involving changing weather conditions (Lu et al., 2025; Farley et al., 2021; Morell et al., 2025; Prova et al., 2022; Grbić and Koch, 2023), whereas others address specific recognition tasks, including vehicle and license plate identification (Aswath et al., 2025; Kaur et al., 2021; Tang et al., 2024). Other datasets are tailored for occupancy detection through bounding box annotations (Balusamy et al., 2024; Nguyen and Sartipi, 2025) or explicit parking-space availability labels (Rafique et al., 2023). This heterogeneity reflects the range of objectives and operating conditions explored in the literature, while also limiting the extent to which performance results can be directly compared between studies.

**Table 20:** Key characteristics of the datasets used.

Key characteristic	Sources
Varied weather conditions (sunny, rainy, cloudy, etc.)	Lu et al., 2025; Farley et al., 2021; Morell et al., 2025; Prova et al., 2022; Grbić and Koch, 2023
License plate/vehicle detection	Aswath et al., 2025; Kaur et al., 2021; Tang et al., 2024
Vehicle tracking and parking bays/occupancy	Nguyen and Sartipi, 2025; Balusamy et al., 2024
Occupied/vacant slot labels	Rafique et al., 2023
Massive, multi-category images (general use)	Duong et al., 2023
Tabulated schedule data (occupancy by hour/day)	Xu et al., 2024
BLE sensors and smartphone data	Park, 2021
People and building occupancy detection	Zhang et al., 2024

Table 21 summarizes the main limitations reported in the analyzed studies. A recurring constraint is the limited environmental diversity of the evaluated datasets (Duong et al., 2023; Balusamy et al., 2024; Farley et al., 2021; Xu et al., 2024; Tang et al., 2024), which may restrict the assessment of model generalization under heterogeneous conditions. Other challenges include performance degradation at night and in variable illumination scenarios (Nguyen and Sartipi, 2025; Lu et al., 2025; Zhang et al., 2024), as well as constraints related to dataset scale and the characteristics of the employed data sources. These limitations highlight persistent challenges regarding the development and evaluation of robust parking detection systems under diverse operational conditions.

**Table 21:** Main limitations reported in the studies analyzed.

Type of limitation	Sources
Lack of climatic or environmental diversity (night, rain, etc.)	Duong et al., 2023; Balusamy et al., 2024; Farley et al., 2021; Xu et al., 2024; Tang et al., 2024
Limitations in night vision or illumination	Nguyen and Sartipi, 2025; Lu et al., 2025; Zhang et al., 2024
Small scale or reduced amount of data	Aswath et al., 2025; Kaur et al., 2021; Park, 2021; Zhang et al., 2024
Bias by location or camera type	Morell et al., 2025; Prova et al., 2022; Grbić and Koch, 2023
Non-specific or generalist datasets	Rafique et al., 2023

Dataset accessibility is an important factor influencing the reproducibility of the reviewed studies. Among the 15 analyzed studies, ten (66.7%) employed publicly available benchmarks, mainly PKLot and CNRPark-EXT, which facilitated replication and comparison across approaches. Conversely, five studies (33.3%) relied on private, context-specific datasets (Aswath et al., 2025; Kaur et al., 2021; Xu et al., 2024; Tang et al., 2024; Park, 2021), generally collected in single locations or controlled environments and, in some cases, with limited sample sizes ranging from 160 images to a few hundred records.

The use of non-shared datasets introduces reproducibility limitations, as external verification of reported results becomes difficult and the restricted diversity of these data sources may affect the interpretation of model generalization. Moreover, the frequent reuse of public benchmarks such as PKLot and CNRPark-EXT highlights a complementary limitation: dataset availability does not necessarily imply broad environmental representativeness, particularly when evaluations are concentrated on similar viewpoints or operating conditions. Studies using private datasets should report acquisition procedures, camera characteristics, image resolution, class distributions, environmental conditions, data splits, anonymized examples, and access conditions, even if the full dataset cannot be released. Future studies should also focus on release model weights, annotations, and training procedures according to the Findable, Accessible, Interoperable, Reusable (FAIR) data principles.

### 3.5 RQ5: Methodological limitations and future research priorities

Table 22 summarizes the main strategies proposed for improving current parking detection systems. Several studies focus on architectural optimization and computational efficiency (Duong et al., 2023; Balusamy et al., 2024; Prova et al., 2022), reflecting the ongoing challenge of achieving accurate detection while maintaining feasible resource requirements. Other studies explore the integration of heterogeneous sensors and real-time multi-source data (Nguyen and Sartipi, 2025; Kaur et al., 2021; Xu et al., 2024; Rafique et al., 2023) to provide richer environmental information and improve system adaptability. Additional proposals involve architectural extensions, including extra processing modules (Lu et al., 2025; Tang et al., 2024), as well as automated parameter optimization techniques. Together, these approaches indicate an increasing interest in systems that combine efficient computation, adaptive processing, and broader contextual information.

**Table 22:** Suggestions for improvement or future research directions.

Type of suggestion	Sources
Architectural optimization/computational efficiency	Duong et al., 2023; Balusamy et al., 2024; Prova et al., 2022
Integration of sensors, multi-source data, and real-time data	Nguyen and Sartipi, 2025; Kaur et al., 2021; Xu et al., 2024; Rafique et al., 2023
Accuracy improvements through additional modules (CBAM, FPN, etc.)	Tang et al., 2024; Lu et al., 2025
Automation and elimination of manual parameters	Aswath et al., 2025; Grbić and Koch, 2023
Fusion of classical models and deep learning	Farley et al., 2021; Park, 2021
Improvements in data input/preprocessing	Morell et al., 2025
Task fusion (detection + classification)	Zhang et al., 2024

Table 23 presents the principal directions for future research identified in the studies analyzed. The predominant emphasis lies on expanding dataset size and diversity, alongside validating models in real-world environments (Duong et al., 2023; Balusamy et al., 2024; Nguyen and Sartipi, 2025; Farley et al., 2021; Morell et al., 2025; Prova et al., 2022; Grbić and Koch, 2023; Rafique et al., 2023), which is essential for enhancing generalization and robustness. Furthermore, several studies have highlighted the integration of these systems within smart city infrastructures (Balusamy et al., 2024; Nguyen and Sartipi, 2025; Xu et al., 2024; Tang et al., 2024; Rafique et al., 2023), as well as the incorporation of complementary sensors and emerging technologies (Kaur et al., 2021; Park, 2021; Rafique et al., 2023) to improve contextual awareness and system performance.

**Table 23:** Proposed future approaches in the studies analyzed.

Future approach	Sources
Dataset expansion / validation in more real-world environments	Duong et al., 2023; Balusamy et al., 2024; Nguyen and Sartipi, 2025; Farley et al., 2021; Grbić and Koch, 2023; Morell et al., 2025; Prova et al., 2022; Rafique et al., 2023
Integration with urban systems / smart cities	Balusamy et al., 2024; Nguyen and Sartipi, 2025; Rafique et al., 2023; Xu et al., 2024; Tang et al., 2024
Use of additional sensors or complementary technologies	Kaur et al., 2021; Park, 2021; Rafique et al., 2023
Application in complex conditions (weather, lighting, obstacles)	Aswath et al., 2025; Park, 2021; Zhang et al., 2024
Application in other areas / domains	Tang et al., 2024

Table 24 presents the principal improvement strategies identified in the reviewed studies. The most prevalent approaches focus on enhancing system *robustness* to environmental variability, including illumination changes and occlusions (Balusamy et al., 2024; Aswath et al., 2025; Kaur et al., 2021; Tang et al., 2024; Grbić and Koch, 2023; Rafique et al., 2023), and increasing computational and resource *efficiency*, particularly for real-time applications and deployment on constrained devices (Nguyen and Sartipi, 2025; Lu et al., 2025; Park, 2021).

**Table 24:** Classification of studies according to proposed improvements.

Type of Improvement	Articles
Improved robustness under challenging conditions (lighting, occlusion, weather, irregular parking)	Aswath et al., 2025; Kaur et al., 2021; Tang et al., 2024; Grbić and Koch, 2023; Rafique et al., 2023; Balusamy et al., 2024
Model efficiency improvements (latency, energy, labeled data)	Nguyen and Sartipi, 2025; Lu et al., 2025; Park, 2021
Accuracy improvements/reduction of detection errors	Duong et al., 2023; Kaur et al., 2021; Morell et al., 2025; Balusamy et al., 2024
Expansion or refinement of datasets	Prova et al., 2022; Xu et al., 2024
Adjustments to annotation techniques/bounding boxes	Farley et al., 2021

### 3.6 RQ6: Preprocessing and data-augmentation practices during training and inference

Table 25 summarizes the preprocessing techniques used in the studies analyzed. The most common procedures include manual annotation (Duong et al., 2023; Aswath et al., 2025; Zhang et al., 2024; Rafique et al., 2023), image resizing to standardized dimensions such as 640 × 640 pixels (Balusamy et al., 2024; Farley et al., 2021; Grbić and Koch, 2023; Zhang et al., 2024; Rafique et al., 2023), and pixel-value normalization (Balusamy et al., 2024; Xu et al., 2024; Prova et al., 2022; Grbić and Koch, 2023). Several studies also apply data augmentation (Balusamy et al., 2024; Zhang et al., 2024; Rafique et al., 2023) and enhancement techniques, including CLAHE and super-resolution (Duong et al., 2023; Kaur et al., 2021; Morell et al., 2025; Zhang et al., 2024), to improve image representation.

**Table 25:** Preprocessing techniques and related articles.

Category	Techniques/processes mentioned	Related articles
Annotation and labeling	Manual annotation with keypoints, bounding boxes (labelImg), and manual masking	Duong et al., 2023; Aswath et al., 2025; Zhang et al., 2024; Rafique et al., 2023
Resizing	Resize to 640 × 640, standard resize without fixed size	Balusamy et al., 2024; Farley et al., 2021; Grbić and Koch, 2023; Zhang et al., 2024; Rafique et al., 2023
Data normalization	Intensity or value normalization	Balusamy et al., 2024; Xu et al., 2024; Prova et al., 2022; Grbić and Koch, 2023
Data augmentation	Implicit augmentation, transfer learning with augmentation	Balusamy et al., 2024; Zhang et al., 2024; Rafique et al., 2023
Filtering/Denoising	Bilateral filtering, median blur, CLAHE, non-local means, and median filter	Duong et al., 2023; Kaur et al., 2021; Morell et al., 2025
Morphological processing	Edge detection (Canny), Hough, morphology (dilatation, erosion, and filling)	Lu et al., 2025; Kaur et al., 2021; Xu et al., 2024
Image enhancement	CLAHE, super-resolution, auto-orientation	Morell et al., 2025; Zhang et al., 2024
Segmentation and ROI	Slot cropping, manual or automatic ROI	Farley et al., 2021; Prova et al., 2022; Grbić and Koch, 2023; Rafique et al., 2023
Tracking	Successive IoU between frames	Nguyen and Sartipi, 2025
OCR and Text extraction	Tesseract OCR	Aswath et al., 2025
Sensor preprocessing/RSSI	Averaging, denoising, and BLE sensor selection	Park, 2021
Format conversion	Conversion to TensorFlow Lite	Aswath et al., 2025

Table 26 summarizes the main categories of preprocessing techniques identified in the analyzed studies. Filtering techniques represent the most frequent approach, appearing in 11 articles (Balusamy et al., 2024; Nguyen and Sartipi, 2025; Aswath et al., 2025; Farley et al., 2021; Kaur et al., 2021; Xu et al., 2024; Park, 2021; Morell et al., 2025; Grbić and Koch, 2023; Zhang et al., 2024; Rafique et al., 2023), mainly targeting improvements in image quality before model processing. Image transformations, including resizing, segmentation, and cropping, are also widely adopted, appearing in 10 studies (Farley et al., 2021; Kaur et al., 2021; Xu et al., 2024; Tang et al., 2024; Park, 2021; Morell et al., 2025; Prova et al., 2022; Grbić and Koch, 2023; Zhang et al., 2024; Rafique et al., 2023).

**Table 26:** Functional classification of preprocessing techniques.

General technique	Frequency	Articles
Augmentation	2	Morell et al., 2025; Zhang et al., 2024
Filtering	11	Balusamy et al., 2024; Nguyen and Sartipi, 2025; Aswath et al., 2025; Farley et al., 2021; Kaur et al., 2021, Xu et al., 2024; Park, 2021; Morell et al., 2025; Grbić and Koch, 2023; Zhang et al., 2024, Rafique et al., 2023
Transfer	2	Zhang et al., 2024; Rafique et al., 2023
Transformation	10	Farley et al., 2021; Kaur et al., 2021; Xu et al., 2024; Tang et al., 2024; Park, 2021, Morell et al., 2025; Prova et al., 2022; Grbić and Koch, 2023; Zhang et al., 2024, Rafique et al., 2023

Table 27 classifies the analyzed studies according to the functional role of data processing in intelligent parking systems.

**Table 27:** Main processing functions in intelligent parking systems.

Category	Main technical function	Related articles
Image or data preprocessing	Input quality improvement (normalization, denoising, and segmentation)	Lu et al., 2025; Park, 2021; Morell et al., 2025; Farley et al., 2021; Kaur et al., 2021; Prova et al., 2022
Parking space detection	Automatic slot localization using computer vision (YOLO, bird's-eye views)	Duong et al., 2023; Nguyen and Sartipi, 2025; Grbić and Koch, 2023; Xu et al., 2024; Tang et al., 2024
Occupancy classification and prediction	Binary classification (occupied/vacant) and predictive models (LSTM)	Rafique et al., 2023; Zhang et al., 2024; Xu et al., 2024; Prova et al., 2022
License plate and text recognition	OCR applied to vehicle license plates	Aswath et al., 2025; Kaur et al., 2021; Tang et al., 2024
Model optimization and robustness	Improving accuracy, efficiency, generalization, and training	Balusamy et al., 2024; Zhang et al., 2024; Rafique et al., 2023

## 4 Discussion

### 4.1 Comparison with previous studies

The findings of this review align closely with Wong et al. (2023) in the dominance of object detection, particularly YOLO. Differences emerge in secondary categories: Wong et al. report 31% segmentation use vs. 2/15 studies here, likely reflecting annotation burden. Direct regression methods identified by Wong et al. are absent; recent studies favor hybrid architectures combining bounding-box detection with sequential mechanisms (Nguyen and Sartipi, 2025; Xu et al., 2024). Both reviews confirm that GNNs remain marginal (Grbić and Koch, 2023).

Both this review and Wong et al. highlight YOLO's prominence. As quantified above, YOLO appears in 46.7% of the corpus—the most frequent family but not an absolute majority. The predominance of YOLO-based models cannot be attributed to inherent architectural superiority because no controlled comparisons with alternative model families were identified. The observed adoption of YOLO may also reflect open-source availability, implementation maturity, extensive documentation, pretrained weights, and broad community support. Performance comparisons cited from Wang et al. (2025) (e.g., 83% and 84.5% mAP) are contextual background, not primary synthesis findings; they situate the corpus within the broader literature without being treated as equivalent to results extracted from the 15 synthesized studies.

A comparison with de Almeida et al. (2022) confirms the reliance on PKLot and CNRPark-EXT. Both reviews attribute this to accessible annotations and high resolution, but also identify limited environmental diversity, restricted viewpoints, and absent nighttime scenarios as shared weaknesses. The reproducibility analysis in the Results section (RQ4) indicates that several studies rely on non-shared or partially documented datasets, limiting independent validation.

Environmental quantification (Table 14) makes the robustness gap concrete: shadows 60%, rain 47%, night 40%, fog 6.7%, occlusions 13.3%, and snow 0%. This imbalance indicates that adverse weather and obstruction scenarios are systematically underrepresented and, not merely acknowledged as future-work items. For smart-city deployment, a system that fails under fog, heavy occlusion, or snowy conditions offers limited operational reliability regardless of its controlled-environment mAP.

The lack of standardized metric reporting—53% of studies omit FPS, 27% omit mAP—undermines comparative assessment. Without reported hardware, input resolution, batch size, and inference settings, FPS values cannot be interpreted reliably for edge deployment or compared across different studies.

### 4.2 Practical impact and deployment barriers

Despite the high controlled-environment accuracy reported for several models (mAP > 90%), the pathway from laboratory performance to large-scale urban deployment involves several barriers that this review's synthesis makes explicit. First, limited environmental validation creates uncertainty regarding operational reliability, maintenance requirements, and deployment costs. Insufficient validation under rain or low illumination prevents reliable estimation of recalibration needs, intervention frequency, and operational availability. Second, privacy compliance requirements (e.g., GDPR) impose architectural constraints: on-device anonymization and edge inference must be integrated into the deployment pipeline without degrading real-time performance. Third, integration with legacy infrastructure—existing traffic management systems, barrier controllers, and payment platforms—adds technical complexity that is not captured by accuracy metrics alone. Future field studies should report guidance accuracy, false-availability events, driver search time, system uptime, intervention frequency, maintenance burden, energy use, and cost per monitored space. Addressing these barriers is necessary to determine whether experimental performance can be translated into reliable operational and societal benefits.

### 4.3 Limitations of this review

This review has the following limitations which should be considered when interpreting its findings.

**Corpus size**. The final corpus comprised 15 primary studies. Its size reflects the eligibility and quality criteria, selected databases, journal-focused scope, English-language restriction, and temporal window; therefore, relevant studies may remain outside this corpus. The review prioritized studies that met the predefined methodological threshold, but this decision may have reduced the diversity of methods and evaluation scenarios represented in the synthesis. The corpus could be expanded in future updates by: (a) including IEEE Xplore as a standalone database; (b) relaxing CE03 to admit high-quality surveys as contextual evidence; and (c) adopting a living systematic-review format given the rapid pace of development in this domain.

**IEEE Xplore exclusion**. IEEE Xplore was not searched as a standalone source. This may have excluded conference papers and IEEE-specific records, including early-stage developments in this rapidly evolving field. Therefore, the review should therefore be interpreted as primarily journal-focused. Future extension should systematically query IEEE Xplore and compare the additional records with the present corpus.

**Protocol registration**. The protocol was not prospectively registered, which limits the independent verification of the original methodological decisions and subsequent amendments.

**Database and language scope**. The review included only Web of Science, ScienceDirect, and Dimensions, and only English publications. Relevant studies indexed in other repositories or published in other languages may have been excluded from this review.

**Temporal scope**. The literature search was updated until March 2026. Studies published or indexed after this period were not included. Because deep learning architectures evolve rapidly, the synthesis may become outdated as new models and datasets appear after March 2026.

**Quality assessment limitations**. The original six-criterion rubric was amended during the revision by adding CC07. Because this amendment was not prospectively registered, it should be considered when interpreting quality-based selection. The absence of code or model weights in primary studies also limits the extent to which true methodological reproducibility can be verified through a review.

**Evidence heterogeneity**. Differences in datasets, train–test splits, IoU thresholds, hardware, input resolution, annotation units, and metric definitions prevented a valid quantitative meta-analysis and limited direct cross-study comparison.

**Publication bias**. This review may over-represent studies with positive results, as null or negative findings are less likely to reach indexed journals within the defined temporal window.

**Reviewer judgment**. Although predefined categories guided data extraction, decisions concerning method classification, environmental-condition coding, and reporting completeness involved interpretive judgment. The consensus review reduced, but did not eliminate, this source of bias.

## 5 Conclusions

The reviewed evidence shows that object-detection methods, particularly YOLO variants, are the most frequently adopted method in recent parking-detection research. YOLO-based approaches appeared in seven of the 15 studies; however, the absence of controlled head-to-head comparisons with transformer-, segmentation-, and graph-based alternatives prevents conclusions about universal architectural superiority.

Several studies have reported mAP values above 90% and processing speeds above 40 FPS. However, differences in datasets, hardware, input resolution, metric definitions, and experimental protocols substantially limit direct comparisons. Missing mAP, FPS, and hardware information further constrain the conclusions about computational efficiency and scalability.

Environmental evaluation remains inconsistent. Fog and occlusion have rarely been examined, and snow is absent from the reviewed evaluations. Therefore, real-world robustness across diverse climates and operating conditions remains insufficiently demonstrated rather than being disproven.

Public benchmarks such as PKLot and CNRPark-EXT remain central to the field, but the repeated use of similar viewpoints and conditions limits environmental representativeness. Private and mixed datasets, along with incomplete implementation reporting, restrict external validation and reproducibility.

This review contributes an analytical performance matrix (Table 13) and a quantified environmental evaluation-coverage framework (Table 14) that identifies recurring gaps in reporting and validation. Future studies should conduct standardized comparisons between YOLO, transformer-based, segmentation-based, and graph-based models using identical data splits, hardware, resolutions, and metrics; develop openly available datasets covering night, fog, occlusion, snow, and other extreme conditions; report a minimum set of accuracy and efficiency measures with full computational context; and release code, model weights, annotations, and metadata whenever possible. Longitudinal field evaluations should also measure operational outcomes such as guidance accuracy, system availability, intervention burden, energy consumption, and cost per monitored space.

Preprocessing and data augmentation are widely used across the reviewed studies, but their individual contributions should be established through controlled ablation analyses rather than assumed from overall model performance.

## Data Availability

The original contributions presented in the study are included in the article/supplementary material, further inquiries can be directed to the corresponding author.
